# Subjecting Dams to Early Life Stress and Perinatal Fluoxetine Treatment Differentially Alters Social Behavior in Young and Adult Rat Offspring

**DOI:** 10.3389/fnins.2019.00229

**Published:** 2019-03-12

**Authors:** Danielle J. Houwing, Laura Staal, Judith M. Swart, Anouschka S. Ramsteijn, Markus Wöhr, Sietse F. de Boer, Jocelien D. A. Olivier

**Affiliations:** ^1^Behavioural Neuroscience Unit, Neurobiology Department, Groningen Institute for Evolutionary Life Sciences, University of Groningen, Groningen, Netherlands; ^2^Experimental and Biological Psychology Department, University of Marburg, Marburg, Germany

**Keywords:** SSRI, development, serotonin transporter, ultrasonic vocalizations, behavior

## Abstract

Recently, the putative association between selective serotonin reuptake inhibitor (SSRI) exposure during pregnancy and the development of social disorders in children has gained increased attention. However, clinical studies struggle with the confounding effects of maternal depression typically co-occurring with antidepressant treatment. Furthermore, preclinical studies using an animal model of maternal depression to study effects of perinatal SSRI exposure on offspring social behavior are limited. Therefore, the aim of this study was to investigate effects of perinatal fluoxetine exposure on juvenile and adult social behavior in male and female rat offspring, using an animal model of maternal vulnerability. We exposed heterozygous serotonin transporter (SERT) deficient female rats to early life maternal separation stress, and used this as a model for maternal vulnerability. Control and early life stressed heterozygous serotonin transporter knockout (SERT) dams were treated with the SSRI fluoxetine or vehicle throughout gestation and lactation. Subsequently, both male and female wildtype (SERT^+/+^) and heterozygous (SERT^+/-^) rat offspring were tested for pup ultrasonic vocalizations (USVs), juvenile social play behavior and adult social interaction. Fluoxetine treatment of the dams resulted in a reduced total USV duration in pups at postnatal day 6, especially in SERT^+/+^ males. Perinatal fluoxetine exposure lowered social play behavior in male offspring from both control and early life stressed dams. However, in females a fluoxetine-induced reduction in juvenile play behavior was only present in offspring from control dams. Offspring genotype did not affect juvenile play behavior. Despite fluoxetine-induced behavioral effects at juvenile age, fluoxetine reduced male adult social behavior in offspring from control dams only. Effects of fluoxetine on female adult social behavior were virtually absent. Interestingly, early life stress in dams increased adult social exploration in vehicle exposed SERT^+/+^ female offspring and total social behavior in fluoxetine exposed adult SERT^+/-^ male offspring. Furthermore, SERT^+/-^ males appeared less social during adulthood compared to SERT^+/+^ males. Overall, the present study shows that chronic blockade of the serotonin transporter by fluoxetine during early development has a considerable impact on pup USVs, juvenile social play behavior in both male and female offspring, and to a lesser extent on male social interaction in adulthood.

## Introduction

During pregnancy, about 7–13% of women are depressed or suffer from depressive symptoms (Bennett et al., 2004). More important, up to 5% of pregnant women suffer from major depression and cannot function normally without antidepressant treatment (Gaynes et al., 2005; Melville et al., 2010). SSRIs are the most widely prescribed antidepressants during pregnancy, as they are considered relatively safe for both mother and child (Gentile, 2005). However, SSRIs are able to cross the placenta and can be found in breast milk, thus reaching the developing child (Heikkinen et al., 2003; Noorlander et al., 2008).

Both exposure to a maternal depression as well as SSRIs during pregnancy can influence the child’s development and behavior (Olivier et al., 2013). As a result, there is an ongoing debate about whether the benefits of treating the mother’s depression during pregnancy and the postpartum period outweigh the potential health risks for the child. Recently, both maternal mood and SSRI treatment have been increasingly linked to changes in social development in children. When exposed to SSRIs *in utero* and the early postnatal period, children are at an increased risk for developing Autism Spectrum Disorder (ASD) traits (Gentile, 2015; Man et al., 2015). However, when properly controlled for maternal mood and stress, this link is not as evident (Brown et al., 2017; Yamamoto-sasaki et al., 2019). Also, independent of SSRI treatment, boys are three to four times more likely to get diagnosed with ASD than girls (Christensen et al., 2016). Unfortunately, the long-term impact of the complex interaction between maternal depression and perinatal SSRI treatment on social behavior in the offspring is not fully understood yet.

Animal studies are often used to investigate SSRI treatment effects on offspring development. However, studies looking into the effects of SSRIs on social behavior in the offspring are limited. Like clinical studies, these few animal studies similarly indicate social alterations in the offspring. For example, both pre- and early postnatal SSRI treatment can decrease social play behavior in rodent offspring (Olivier et al., 2011; Simpson et al., 2011; Khatri et al., 2014), or time spent exploring a novel conspecific (Zimmerberg and Germeyan, 2015). In contrast, an increase in aggressive play behavior is also reported (Gemmel et al., 2017). Furthermore, prenatal SSRI treatment can affect social communication in 10-day-old rat pups by increasing the amount of 40-kHz distress calls, reflecting an increase in anxiety (Cagiano et al., 2008). Even so, more limited are animal studies that also take the maternal depression into account. Such studies are more translational to the human situation since SSRI treatment typically occurs when suffering from anxiety and/or depression. In fact, clinical studies indicate that maternal illness is a confounding factor that can drive the risk of ASD in the offspring rather than the SSRI itself (Andrade, 2017). Furthermore, SSRI treatment in healthy pregnant women is considered unethical, but this problem can be overcome in preclinical studies. Consequently, preclinical animal studies investigating both the maternal depression and SSRI treatment separately as well as combined would be more relevant.

To model a maternal depression, studies often utilize dams that were stressed during the gestational or pre-gestational period. Recently, it has been shown that treatment with the SSRI fluoxetine can prevent reductions in rat juvenile social play behavior caused by pre-gestational maternal stress (Gemmel et al., 2017). While these results suggest a protective role of FLX against the effects of pre-gestational maternal stress on juvenile play behavior with siblings, they also show an increase in aggressive play behavior with unfamiliar conspecifics. It still remains to be established whether altered social behavior in the offspring due to maternal stress and/or FLX treatment in the dams remains present during adulthood. Furthermore, the long-term effects of perinatal SSRI use combined with maternal stress on social interactions need to be established in both male and female offspring, as such rodent studies including both sexes are still limited. In addition, sex differences are present in disorders related to social withdrawal (Christensen et al., 2016).

The aim of the present study was to assess the developmental effects of MS and perinatal FLX exposure, both separately and combined, on juvenile and adult social behavior in male and female rat offspring. In addition, pup USVs were investigated. We used female rats heterozygous for the serotonin transporter (SERT^+/-^), and subjected them to early life stress as a translational model for maternal vulnerability, since we have previously shown that this induces anhedonia, which is a well-known symptom of depression (Houwing et al., 2019). Consequently, our dams produce wildtype and heterozygous SERT offspring, so possible effects of SERT genotype on offspring social behavior could be investigated. In particular, FLX treatment was applied during the gestational and postpartum (lactation) period, since rat brain neurodevelopment at PNDs 1–10 is equal to the third trimester in humans (Dobbing and Sands, 1979; Andrews and Fitzgerald, 1997). Furthermore, many women using antidepressants during pregnancy continue using them during the postnatal period. In addition, both male and female offspring were investigated, as there is a lack of animal studies investigating both sexes. We hypothesized that perinatal FLX exposure would result in an increase in 40-kHz distress calls after being separated from the mother and littermates (Cagiano et al., 2008). Furthermore, we expected decreased juvenile social play behavior and adult social interaction, as shown in previous studies (Olivier et al., 2011; Simpson et al., 2011; Khatri et al., 2014), with no differences between genotypes. Even though the dams, and not the offspring, were exposed to the early life stress, we also suspect a possible interaction of offspring genotype with MS on offspring social behavior. We also expected decreased social play behavior in the offspring due to MS as shown previously by Gemmel et al. (2017). Finally, we expected FLX to alleviate effects of MS on the offspring of both sexes (Gemmel et al., 2017). With this study, we want to provide a more complete understanding of the potential long-term risks SSRI treatment during pregnancy and lactation can have on offspring social behavior both early in life as well as later during adulthood.

## Materials and Methods

### Animals

SERT knockout rats (Slc6a41Hubr) were bred crossing SERT^+/-^ females with SERT^+/-^ male rats (F0), resulting in offspring (F1) of three genotypes (SERT^+/+^, SERT^+/-^, and SERT^-/-^). Females were checked daily at 9:00 and 17:00 for delivery of pups, which was set as PND0. Animals were housed in aspen wood chip filled Makrolon type 3 cages (38.2 × 22.0 × 15.0 cm; individual housing) or Makrolon type 4 cages (55.6 × 33.4 × 19.5 cm; social housing) with *ad libitum* access to food (RMH-B, AB Diets; Woerden, Netherlands) and tap water. For enrichment, animals were housed with a wooden gnawing stick (10 × 2 × 2 cm) and nesting material (Enviro-dri^®^) for nest building. Animals were housed under standard laboratory conditions with a reversed 12 h:12 h light/dark cycle (lights off at 11:00 AM). All breeding took place in our own facility. All experimental procedures were approved by the Groningen University Committee of Animal experiments.

### Maternal Early Life Stress

As an early life stressor, pups were subjected to maternal separation early in life. Dams (F0) with pups (F1) were randomly assigned to either the control group (CTR) or the maternal early life stress group (MS). From PND2–15 pups were maternally separated as a whole litter for 6 h a day (9:00 AM to 3:00 PM) and transported to an empty room, to prevent olfactory and visual communication as well as USVs. During maternal separation, the whole litter was placed in a closed Makrolon type 2 cage on a heating mat (Terra Heatmat, Heatel; Poeldijk, Netherlands). In the first postnatal weeks, rat pups are unable to sufficiently regulate their body temperature, as they lose more body heat than they generate (Alberts, 1978) and thus need extra heating elements when absent from the mother for a long period. The temperature of the heating mat was set at 32 ± 1°C during PND2–8, and 28 ± 1°C during PND9–15. CTRs were handled for 15 min in the same room as the dams (e.g., weighing or determining sex) and were not placed on a heating mat. When returned to the mother, a bit of sawdust was distributed on the pups to apply scent of the mother. At PND21 pups were weaned and ears were punched for identification and genotyping (genotyping procedure: El Aidy et al., 2017). When adult, CTR and MS SERT^+/-^ female offspring (F1) were used as dams for the next part of the study. To validate our animal model of depression, the dams were tested for depressive-like behavior (Houwing et al., 2019). SERT^+/-^ females exposed to maternal separation showed a lower preference for sucrose compared to CTR SERT^+/-^ females, reflecting anhedonic behavior. In addition, it was found that MS SERT^+/-^ females have significantly less nerve growth factor gene expression in the basolateral amygdala and paraventricular nucleus of the hypothalamus compared to CTR SERT^+/-^ females (Houwing et al., 2019). These observed reductions in nerve growth factor levels may contribute to the underlying mechanisms of depressive-like behavior found in these dams.

### Perinatal Fluoxetine Exposure

In total, 85 adult female SERT^+/-^ (33 CTR and 52 MS) and 47 male SERT^+/+^ Wistar rats were used for breeding (F1). The estrus cycle phase of the females was determined by measuring vaginal wall impedance (model MK-11, Muromachi, Tokyo, Japan). When in estrus, a female was placed with a male for a 24-h period (Gestational day 0: G0) before removing the male. Females from both CTR and MS groups were randomly assigned to the FLX or VEH group, with more dams assigned to the FLX groups. From G0 until weaning of the pups at PND21 (for 6 weeks), dams were weighed and treated daily at 11:00 AM with either 10 mg/kg FLX (Fluoxetine 20 PCH, Pharmachemie B.V., Netherlands) or a VEH (methylcellulose 1%, Sigma-Aldrich Chemie B.V., Zwijndrecht, Netherlands) using oral gavage. Methylcellulose was used as this is the constituent of the FLX capsule. For this procedure, flexible PVC feeding tubes normally used for baby enteral tube feeding (40 cm length, Vygon, Valkenswaard, Netherlands) were used. Using these feeding tubes, animals can be orally treated by gently picking up the animal without restraining them. By doing so we sought to minimize stress. Even so, animals injected with FLX, but not VEH, appeared to struggle more after repeated oral treatment. Four groups of dams were used: (1) CTR dams (no MS) + VEH treatment (CTR-VEH) (*n* = 11), (2) CTR dams (no MS) + FLX treatment (CTR-FLX) (*n* = 22), (3) MS + VEH treatment (MS-VEH) (*n* = 15) and (4) MS + FLX treatment (MS-FLX) (*n* = 37). Near the end of pregnancy, dams were checked twice a day (9:00 and 17:00) for pup delivery. On the day of birth (PND0), when the dam was taken out of the cage for her daily treatment, the birth weight and number of live and dead pups was recorded. In addition, the number of live pups was counted daily until PND4. At PND21, offspring (F2) were weaned and ears were punched for individual recognition and genotyping. Both wildtype (SERT^+/+^) and heterozygous (SERT^+/-^) offspring were used in this study. Pups were housed in groups of three to five with same sex and same treated animals in Makrolon type 4 cages under the same conditions as the dams. Pups were weighed weekly from PND0 until PND70. Due to unexpected high mortality rates in dams and offspring from FLX groups, three batches were needed to complete the study.

### Pregnancy Outcomes

During gestation and lactation, the following data were recorded of all three batches unless stated otherwise: dam mortality, gestational length, live birth index ([number of live offspring/number of offspring delivered] ^∗^ 100), the viability index ([number of live offspring on PND4/number of live offspring delivered] ^∗^ 100), and litter size at birth and weaning. Furthermore, pup weight at birth and at PND70 were shown (batch 3 only).

### Ultrasonic Vocalizations (USVs)

At PND6, pup USVs in response to separation from the dam and littermates were recorded (batch 3 only) (Wöhr and Schwarting, 2008). In short, dams with litters on PND6 were moved to an empty room adjacent to the test room at least half an hour prior to recordings. Wearing gloves, one pup was randomly selected, sex determined and moved individually to the test room. No other animals were present in the test room. The pup was placed in a Makrolon type 2 cage (22.5 × 16.7 × 14 cm) filled with Aspen wood chip that was already under the ultrasonic microphone (Condenser ultrasound microphone Avisoft-Bioacoustics CM16/CMPA, Glienicke, Germany). Recording started immediately and lasted for 3 min. Using the software Avisoft-SASLab Pro (version 5.2.12, Avisoft Bioacoustics, Glienicke, Germany) the time of the onset and offset of each vocalization was recorded, allowing for the determination of the total USV duration. Since genotype was yet unknown, pups were marked afterward with a black marker on one of their paws for individual recognition. From each dam’s litter, a maximum of two pups per sex and genotype were analyzed. Since the USV experiment was run as a pilot, we only recorded USVs from pups from CTR (VEH and FLX) dams and not pups from dams exposed to early life stress. In total 32 males (9 SERT^+/+^ VEH, 11 SERT^+/+^ FLX, 5 SERT^+/-^ VEH, 7 SERT^+/-^ FLX) and 33 females (7 SERT^+/+^ VEH, 9 SERT^+/+^ FLX, 8 SERT^+/-^ VEH, 9 SERT^+/-^ FLX) were recorded. All recordings took place between 2:30 and 5:00 PM under dim-light conditions.

### Behavioral Testing

Male and female SERT^+/+^ and SERT^+/-^ offspring from the four different dam groups were assessed for juvenile play behavior and social interaction during adulthood. This resulted in 16 offspring groups (Table 1). In general one to three pups per sex and genotype per litter were used. However in 4.7% of the litters we used four to five pups per litter because of the reduced survival of pups, but did not find significant litter effects. The number of litters used for the offspring group is represented in Table 1. Testing occurred during the active dark phase of the animals, between 12:00 and 5:00 PM.

**Table 1:** All 16 different offspring groups from four groups of dams that underwent behavioral assessment.

Dams		Offspring		Juvenile social play (*n*)^∗^	Adult social interaction (*n*)	No. of litters used
CTR	VEH	Male	SERT^+/+^	22	12	10
			SERT^+/-^		10	10
		Female	SERT^+/+^	24	11	11
			SERT^+/-^		11	11
CTR	FLX	Male	SERT^+/+^	20	10	15
			SERT^+/-^		10	10
		Female	SERT^+/+^	21	11	12
			SERT^+/-^		10	11
MS	VEH	Male	SERT^+/+^	26	13	12
			SERT^+/-^		13	11
		Female	SERT^+/+^	23	12	9
			SERT^+/-^		11	11
MS	FLX	Male	SERT^+/+^	24	12	15
			SERT^+/-^		10	13
		Female	SERT^+/+^	23	9	15
			SERT^+/-^		11	16


*Each ‘*n*’ depicts the number of couples used. ^∗^For social play analysis SERT^+/+^ and SERT^+/-^ offspring were grouped. The last column shows the number of animals each offspring group came from.*

#### Social Play

Offspring were tested for juvenile social play behavior at 4–5 weeks of age (PND28–35), when the occurrence of this behavior is at its peak (Panksepp, 1981; Pellis and Pellis, 1991). Animals were tested in couples, with a sex, genotype and treatment matched unfamiliar play partner, since play behavior in rats depends on the playfulness of the partner (Varlinskaya et al., 1999). The age of the test partners did not differ more than 1 week. Prior to testing, motivation to play was increased to half maximum by physically isolating the animals for 3.5 h (Niesink and Van Ree, 1989). During isolation, individuals were housed in Makrolon type 3 cages with *ad libitum* access to tap water and food. Animals were marked on their tail for individual recognition. Furthermore, animals were habituated individually to the test cage for 5 min during the isolation period. During testing, couples were placed in wood chip filled Phenotyper cages (45 × 30 × 50 cm, Noldus Information Technology B.V., Wageningen, Netherlands) for 15 min under red light conditions. Play behavior was videotaped from above using an infrared camera in the Phenotyper top unit. Multiple couples in different cages were tested at the same time, but were not visible to each other. Various social play behaviors were scored including time spent socially exploring the partner (sniffing or grooming), pouncing frequency, pinning frequency, chasing duration and boxing/wrestling duration (Trezza et al., 2011). To get an idea about the total time spent in social play behavior, pouncing, pinning, chasing and boxing/wrestling durations were combined to ‘total play behavior.’ Each couple was considered as an experimental unit, scored by a blind observer and analyzed using The Observer XT version 11.0 (Noldus Information Technology B.V., Wageningen, Netherlands). Social play behavior was scored when at least one of the two animals was displaying the behavior (e.g., when rat 1 is pouncing the behavior is scored, but also when rat 2 is pouncing).

#### Social Interaction

At 10–11 weeks of age, the same animals were tested for a different social behavior test, namely social interaction. When rats grow up and sexually mature, social play behavior gradually declines but does not disappear entirely (Panksepp, 1981; Pellis and Pellis, 1991). To increase motivation for social interaction, animals were isolated for 48 h prior to testing as done previously (Olivier et al., 2011). Animals were individually habituated to the test cage for 20 min at the beginning of the isolation period and again 20 min when 24 h into the isolation period. After the 48-h isolation period, couples were placed in a rectangular arena (80 × 55 × 40 cm) to socially interact for 15 min under dim light conditions (10 lux). Animals were tested with the same partner as during play behavior. Social interaction was videotaped from the front and duration was scored using The Observer 11.0 (Noldus Information Technology B.V., Wageningen, Netherlands). Scored behaviors include socially exploring the partner (sniffing, licking or grooming); following or approaching the partner; and play behavior (pouncing; pinning). To look at overall social behavior, all scored behaviors were combined into ‘total social behavior.’ Due to technical difficulties during social interaction tests, two couples were not recorded. Furthermore, two couples were tested with the wrong partner, and three couples were not tested because their play partner did not survive into adulthood. Thus, even though the same couples were tested for social play and interaction, some groups have fewer couples for social interaction.

### Statistical Analysis

The Statistical Package for the Social Sciences software version 22 (SPPS Inc., IBM SPSS Statistics, Chicago) was used to perform statistics. Data was checked for parametric distribution and transformed if non-parametric for use in statistical analysis [formula for positively skewed data: Log_10_(behavioral parameter +1), formula for negatively skewed data: Log_10_(max value +1 – behavioral parameter)]. For pregnancy outcomes, the live birth- and viability index were negatively skewed and transformed. For social play the following behavioral parameters were positively skewed: pinning frequency and chasing duration. Boxing/wrestling duration occurred on average less than 2 s per group and was not individually analyzed. For social interaction behavior, total play behavior was found to be positively skewed and was therefore transformed.

Ultrasonic vocalizations were analyzed using a three-way (FLX × genotype × sex) ANOVA to determine main and/or interaction effects. *Post hoc* paired *t*-tests for planned contrasts per sex and genotype were performed. For pregnancy outcomes a two-way (MS × FLX) ANOVA was performed. For behavioral data, first a 4-way multifactorial ANOVA (MS × FLX × genotype × sex) was performed to determine main sex and/or genotype effects (Supplementary Table 1). No main effects of sex were found for social play behaviors. Furthermore, a genotype effect was only found for pinning frequency. Consequently, we decided to group genotypes but split the data by sex anyway for further analysis since it is known that there are sex differences in response to stress and FLX exposure on behavioral measures. Subsequently, a two way (MS × FLX) multifactorial ANOVA per sex was performed to determine main and/or interaction effects. Upon significant main or interaction effects, *post hoc* testing using Fisher’s LSD to correct for multiple comparisons was performed. Furthermore, a 4-way multifactorial ANOVA (MS × FLX × genotype × sex) for adult social interaction showed multiple main effects of sex and genotype, and an interaction between FLX exposure, genotype and sex and genotypes were therefore not collapsed (Supplementary Table 1). As a result, a three way (MS × FLX × genotype) multifactorial ANOVA per sex was performed. In addition, Fisher’s LSD *post hoc* was used to correct for multiple comparisons. All statistics were two-tailed with values of *p* < 0.05 being considered significant. All data and all figures are presented as means ± standard error of the mean (SEM).

## Results

### Pregnancy Outcomes

To examine the effects of MS and FLX treatment on pregnancy outcomes, basic parameters such as dam mortality, gestational length, pup survival (live birth and viability index), litter size at birth and weaning and pup weight at birth and PND70 were recorded. Both FLX exposure and MS affected various pregnancy outcomes (Table 2). Dam mortality differed among dams [*X*^2^_(3,_ *_n_*_= 85)_ = 9.980, *p* < 0.05], with dams treated with FLX having higher mortality (∼30%) than VEH treated dams (0%). FLX treatment of the dams significantly increased gestational length [*F*_(1,70)_ = 8.063, *p* < 0.01], regardless of MS, while MS decreased the gestational length, regardless of FLX treatment [*F*_(1,70)_ = 14.268, *p* < 0.001]. *Post hoc* analysis revealed that dams from the MS-VEH group had a significantly shorter gestation period compared to all other groups (vs. CTR-VEH: *Z* = -2.612, *p* < 0.01; vs. CTR-FLX: *Z* = -4.062, *p* < 0.001; vs. MS-FLX: *Z* = -2.564, *p* = 0.01). The addition of FLX to MS restored the gestational length to CTR length (*Z* = -0.599, *p* > 0.05), but was still shorter than the gestational length from dams treated with FLX only (*Z* = -2.323, *p* < 0.05). The live birth index was significantly decreased after FLX exposure, regardless of MS [*F*_(1,66)_ = 31.198, *p* < 0.001]. Both FLX groups had a significant lower live birth index than VEH treated mothers (CTR-VEH vs. CTR-FLX: *p* < 0.001; MS-VEH vs. MS-FLX: *p* < 0.01). From those born alive, fewer pups from FLX exposed groups survived until PND4 [viability index: *F*_(1,66)_ = 37.482, *p* < 0.001]. Again, FLX exposed pups from CTR dams (*p* < 0.001) as well as from early life stressed dams (*p* = 0.001) had a lower viability index than VEH treated CTR dams. Weight of the pups at PND0 was significantly decreased by FLX treatment, regardless of MS [*F*_(1,233)_ = 28.519, *p* < 0.001]. Similarly, MS significantly decreased birth weight of pups, regardless of FLX treatment [*F*_(1,233)_ = 20.717, *p* < 0.001]. *Post hoc* testing revealed that offspring from CTR dams weighed more than all other groups (CTR-FLX: *Z* = -5.871, *p* < 0.001; MS-VEH: *Z* = -4.706, *p* < 0.001; MS-FLX: *Z* = -7.647, *p* < 0.001). Pups in all groups did not differ in their weight at PND70 (*p* > 0.05) No significant main or interaction effects of treatment were found on litter size at birth. However, FLX exposure significantly affected litter size at weaning [*F*_(1,66)_ = 33.991, *p* < 0.001], decreasing the litter size from both CTR dams (*p* < 0.001) and dams stressed early in life (*P* < 0.001).

**Table 2:** Pregnancy outcomes of dams treated with fluoxetine from G1 to PND21.

	CTR-VEH^a^	CTR-FLX^b^	MS-VEH^c^	MS-FLX^d^
Number of dams	11	22	15	37
Dam mortality (%)	0/11 (0%)^bd^	7/22 (32%)^ac^	0/15 (0%)^bd^	11/37 (30%)^ac^
Gestational length (days)	22.64 ± 0.15^c^	22.88 ± 0.09^cd^	22.13 ± 0.09^abd^	22.50 ± 0.09^bc^
Live birth index (%)	96.7 ± 2.00^bd^	76.9 ± 4.70^ac^	99.0 ± 0.67^bd^	83.8 ± 3.32^ac^
Viability index (%)	98.4 ± 1.1^bd^	71.9 ± 5.9^ac^	97.2 ± 2.3^bd^	73.0 ± 6.0^ac^
Litter size at birth	11.8 ± 0.9	11.7 ± 0.4	12.4 ± 0.7	12.0 ± 0.3
Litter size at weaning	11.2 ± 0.8*^bd^*	7.1 ± 0.5*^ac^*	11.9 ± 0.7*^bd^*	8.0 ± 0.5*^ac^*
Pup birth weight (g)	5.88 ± 0.05^bcd^	5.34 ± 0.05^a^	5.40 ± 0.11^a^	5.26 ± 0.04^a^
Pup weight at PND70 (g)	205.7 ± 4.8	211.7 ± 5.6	202.8 ± 8.0	206.7 ± 4.7


*Letters indicate a significant difference of *p* < 0.05 with the respective group (^*a*^CTR-VEH; ^*b*^ CTR-FLX; ^*c*^ MS-VEH; ^*d*^ MS-FLX).*

### Ultrasonic Vocalizations

**FIGURE 1:**
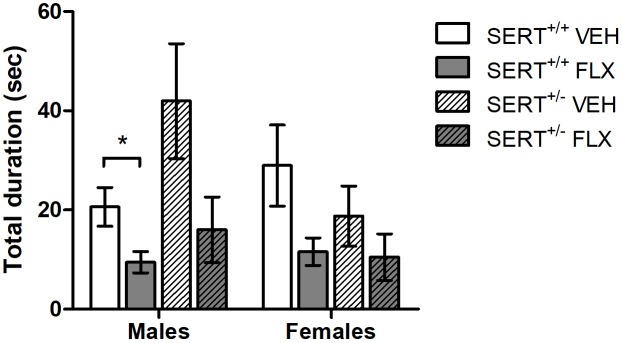
Total USV duration of VEH or FLX exposed pups separated from their dam and littermates on PND6. Shown is the mean ± SEM. ^∗^*p* < 0.05, *n* = 5–9 per group.

Pups exposed to perinatal FLX treatment significantly lowered the total duration of USVs after separation from mother and littermates compared to perinatal VEH exposed pups, regardless of sex or genotype of the pups [*F*_(1,56)_ = 19.406, *p* < 0.001, Figure 1]. No main effects of sex or genotype, or interactions with FLX treatment were found on total USV duration. *Post hoc* analysis showed that SERT^+/+^ males from FLX treated dams had a significant lower total call duration (*t* = 2,627, df = 18, *p* < 0.05) compared to SERT^+/+^ male pups from VEH treated dams. The same reduction in USVs was found in FLX exposed SERT^+/+^ females, however this just missed significance (*t* = 2.010, df = 7.391, *p* = 0.08). Similarly, SERT^+/-^ males from FLX treated dams tended to have a lowered total call duration compared to SERT^+/-^ males from VEH treated dams (*t* = 2.084, df = 10, *p* = 0.06), while no differences were found in SERT^+/-^ females. In addition, a sex × genotype effect was found, regardless of FLX treatment [*F*_(1,56)_ = 8.094, *p* < 0.01]. *Post hoc* analysis showed that SERT^+/-^ males from VEH treated dams tended to have a higher total call duration than SERT^+/+^ males (*p* = 0.05).

### Social Play Behavior

**FIGURE 2:**
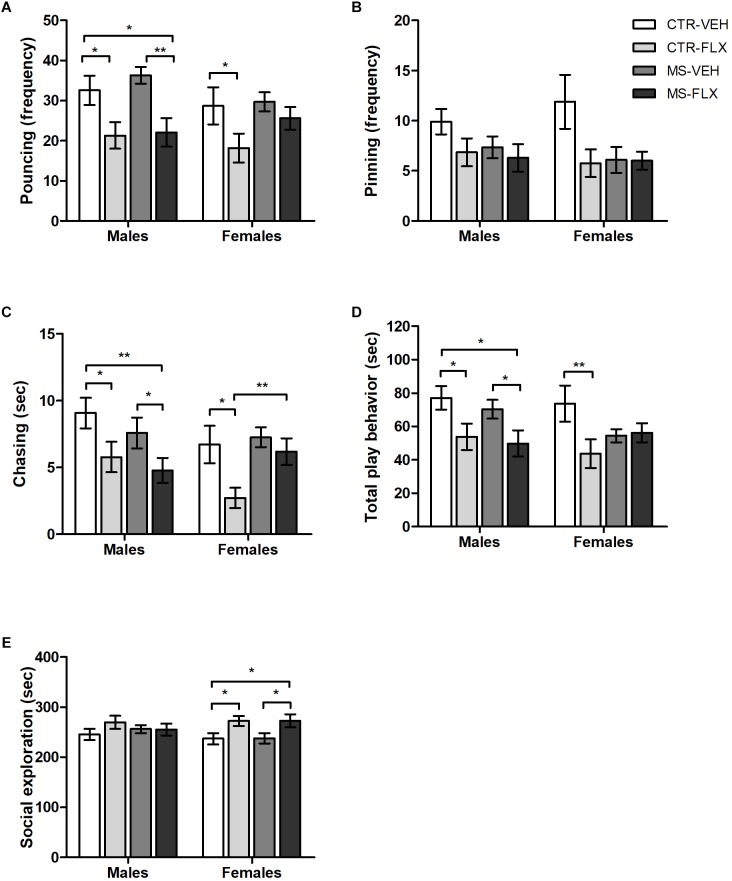
Occurrence of specific play behaviors such as pouncing frequency **(A)**, pinning frequency **(B)**, chasing duration **(C)**, total time spent in play behavior **(D)** and time spent socially exploring the partner **(E)** in male and female juvenile rat offspring exposed to fluoxetine (FLX), maternal early life stress (MS) or their combination. Figures show mean ± SEM. ^∗^*p* < 0.05, ^∗∗^*p* < 0.01. SERT genotypes were collapsed. *n* = 20–26 per group (Table 1).

To assess whether and how MS and FLX exposure affect juvenile play behavior in male and female offspring, we measured social play behaviors with an unfamiliar partner. Since little genotype effects were found, play behavior from SERT^+/+^ and SER^+/-^ animals were collapsed, differentiating only between males and females from the four different treatment groups. Pouncing and pinning are the most characteristic forms of social play behavior and easy to recognize. Both male and female offspring showed a significant reduction in pouncing frequency when exposed to FLX, regardless of MS [males: *F*_(1,88)_ = 18.142, *p* < 0.001; females: *F*_(1,87)_ = 4.403, *p* < 0.05, Figure 2A]. As expected, *post hoc* testing revealed that both male and female CTR-FLX offspring pounced less than CTR-VEH animals (male: *p* < 0.05; female: *p* < 0.05). In addition, there was a significant decrease in pouncing frequency of MS-FLX male offspring compared to CTR-VEH (*p* < 0.05) and MS-VEH (*p* = 0.001) male offspring. Remarkably, no effects of FLX treatment or MS were found on the pinning frequency (Figure 2B). Another play component, chasing duration, was altered after perinatal FLX exposure in both males [*F*_(1,88)_ = 10.008, *p* < 0.01] and females [*F*_(1,87)_ = 6.067, *p* < 0.05, Figure 2C]. *Post hoc* analysis showed a reduction in chasing duration in male (*p* < 0.05) and female (*p* < 0.05) offspring from FLX treated mothers compared to VEH treated mothers. In males, the combination of MS and FLX exposure also decreased offspring chasing duration, compared to both VEH treated groups (CTR-VEH: *p* < 0.01, MS-VEH: *p* < 0.05). However, MS prevented the decrease in chasing behavior caused by FLX exposure in female offspring (*p* < 0.01).

For the total duration of play behavior (including duration of pouncing, pinning, chasing and boxing/wrestling), FLX exposure led to a reduction in social play in male offspring regardless of MS [*F*_(1,88)_ = 8.731, *p* < 0.01, Figure 2D]. *Post hoc* testing showed a significant decrease in male total play behavior of both CTR-FLX (*p* < 0.05) and MS-FLX (*p* < 0.05) compared to CTR-VEH offspring. Furthermore, male MS-FLX offspring played significantly less than MS-VEH offspring (*p* < 0.05). A significant interaction was found between FLX treatment and MS on total play behavior in females [*F*_(1,87)_ = 4.082, *p* < 0.05, Figure 2D]. Further analysis revealed that CTR-FLX females played significantly less than offspring of CTR-VEH mothers (*p* < 0.01). Similarly, MS tended to reduce play behavior in female offspring from VEH-treated mothers (*p* = 0.08).

When it comes to sniffing or grooming an unfamiliar play partner, FLX exposure led to a significant increase in social exploration in female offspring regardless of MS [*F*_(1,87)_ = 9.545, *p* < 0.01, Figure 2E]. Further *post hoc* analysis revealed an increase in female social exploration in both CTR-FLX (*p* < 0.05) and MS-FLX (*p* < 0.05) offspring compared to CTR-VEH. In addition, female MS-FLX offspring also showed an increase in social exploration compared to female MS-VEH offspring (*p* < 0.05). Social exploration in male offspring was not affected by FLX exposure or MS (*p* > 0.05, Figure 2E).

Overall, males appear to be more vulnerable to the effects of FLX when it comes to juvenile play behavior, since FLX exposure both with and without MS reduced play behaviors. However, FLX exposure only reduced play behavior in female offspring from CTR mothers. This FLX-reduced juvenile play behavior was not present in female offspring from early life stressed dams.

### Social Interaction

**FIGURE 3:**
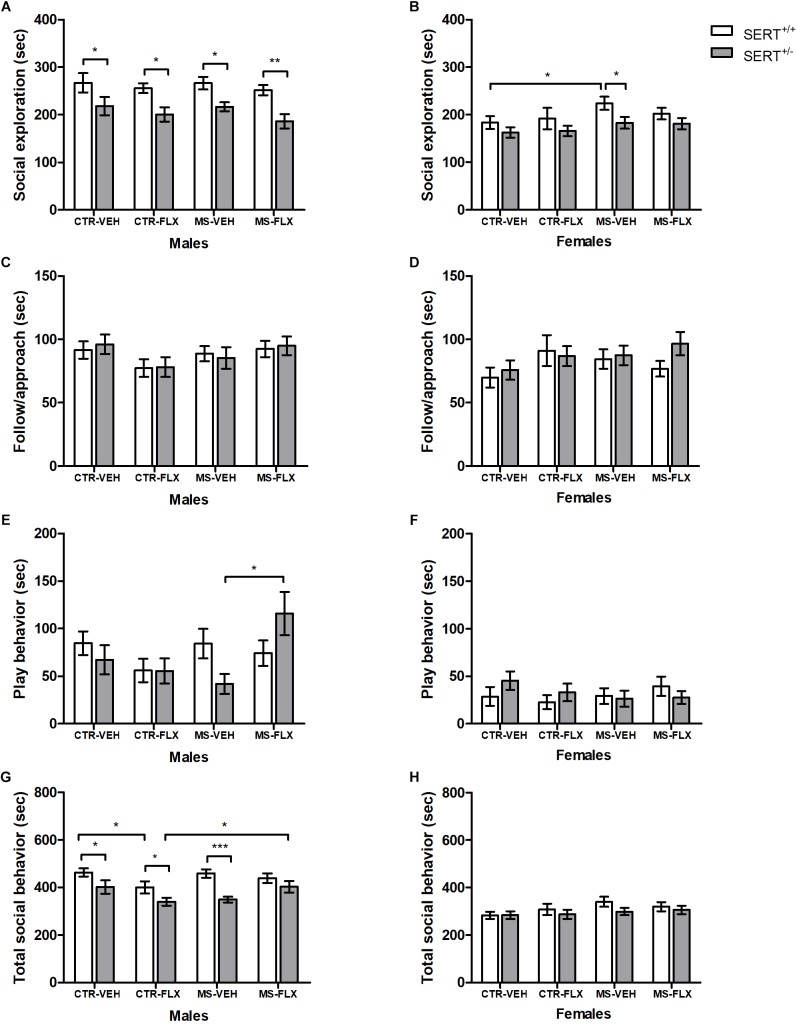
Time spent in adult social interactions such as social exploration **(A,B)**, follow/approach **(C,D)**, play behavior **(E,F)**, and all social behavior combined **(G,H)** in male and female adult offspring exposed to fluoxetine (FLX), maternal early life stress (MS) or their combination. Figures show mean ± SEM. ^∗^*p* < 0.05, ^∗∗^*p* < 0.01, ^∗∗∗^*p* < 0.001. *n* = 9–13 per group (Table 1).

During adulthood, offspring was tested in a different set-up to test social interaction. Adult male and female offspring heterozygous for the SERT showed reduced social exploration toward a conspecific compared to wildtype offspring, regardless of FLX exposure and MS [male: *F*_(1,82)_ = 28.128, *p* < 0.001, female: *F*_(1,78)_ = 7.534, *p* < 0.01, Figure 3A and Figure 3B]. *Post hoc* testing showed that male SERT^+/-^ animals in all four offspring groups spent less time socially exploring their partner compared to wildtypes (CTR-VEH: *p* < 0.05, CTR-FLX: *p* < 0.05, MS-VEH: *p* < 0.05, MS-FLX: *p* < 0.01), while in female offspring this was only the case for SERT^+/-^ MS-VEH offspring (*p* < 0.05). Furthermore, MS led to a significant increase in female offspring social exploration, regardless of genotype and FLX exposure [*F*_(1,78)_ = 4.672, *p* < 0.05]. Further analysis revealed an increase in social exploration in female SERT^+/+^ offspring from the MS-VEH group compared to SERT^+/+^ CTR-VEH offspring (*p* < 0.05).

An interaction was found between MS and FLX exposure on following/approach behavior in male offspring, regardless of genotype [*F*_(1,82)_ = 4.937, *p* < 0.05; Figure 3C]. However, *post hoc* analysis showed no significant effects on follow/approach behavior (*p* > 0.05). In females, no main or interaction effects of MS and FLX exposure were found on offspring following/approach (*p* > 0.05, Figure 3D).

An interaction between MS and FLX exposure in male offspring was found for total time spent in play behavior [*F*_(1,82)_ = 5.250, *p* < 0.05, Figure 3E]. Also, an interaction was found between genotype and FLX exposure on total play duration in male offspring [*F*_(1,82)_ = 4.944, *p* < 0.05]. When analyzed further, male SERT^+/-^ offspring from the MS-FLX group had increased total play behavior compared to male SERT^+/-^ MS-VEH (*p* < 0.05) offspring. No main or interaction effects were found in female offspring (*p* > 0.05, Figure 3F). When looking at total social behavior, an interaction effect of MS and FLX treatment was found in male offspring [*F*_(1,82)_ = 6.979, *p* = 0.01, Figure 3G], but not for females (*p* > 0.05, Figure 3H). *Post hoc* testing showed that CTR-FLX SERT^+/+^ male offspring had significantly decreased total social behavior compared to CTR-VEH SERT^+/+^ offspring (*p* < 0.05). Combining MS and FLX exposure increased total social behavior in male SERT^+/-^ offspring (*p* < 0.05). In addition, a significant main effect of offspring genotype was found, reflecting decreased total social behavior in SERT^+/-^ male offspring compared to SERT^+/+^ animals [*F*_(1,82)_ = 23.590, *p* < 0.001]. Further analysis revealed a reduction of total social behavior in male SERT^+/-^ offspring in CTR-VEH (*p* < 0.05), CTR-FLX (*p* < 0.05) and MS-VEH (*p* < 0.001) groups compared to male SERT^+/+^ offspring.

Overall, effects of FLX exposure found during juvenile play are less apparent during adult social interactions in males, and are completely absent in females, suggesting that males are more vulnerable to the effects of perinatal FLX exposure when it comes to adult social behavior. Furthermore, effects of MS are now present in some offspring groups, with MS increasing offspring social behavior in males and females. Finally, having less SERT gene expression resulted in reduced adult social behavior, an effect that was more prominent in males.

## Discussion

To our knowledge, the present study was the first to assess the effects of MS and perinatal FLX exposure, both separately and combined, on both juvenile and adult social behavior in male and female offspring. In addition, this study is the first to take both SERT^+/+^ and SERT^+/-^ offspring into account. The results demonstrated that perinatal FLX exposure negatively affected pregnancy outcomes and lowered pup USVs. Furthermore, social play behavior of juvenile male and female offspring was affected. Male and female offspring of CTR dams treated with FLX displayed a considerable reduction in various components of social play behavior, as well as in total play behavior, independent of genotype. However, this FLX-reduced juvenile play behavior was not present in female offspring from early life stressed dams. In adulthood, reductions found in juvenile social behaviors due to perinatal FLX exposure are only present in the total time spent in social behaviors in wildtype male offspring. Interestingly, these FLX effects are entirely absent in females from both genotypes. Besides effects of perinatal FLX exposure, MS resulted in increased adult social interaction in male and female offspring, but in some treatment groups only. Overall, heterozygous offspring displayed lower social behavior than wildtype offspring, especially the males.

### Perinatal SSRI Exposure and Pregnancy Outcomes

In the present study, exposing SERT^+/-^ dams to 10 mg/kg FLX during the entire pregnancy and lactation period resulted in high dam mortality (about 30%), suggesting maternal toxicity at the used dose. Timing of mortality was random (sometimes within minutes, other times it took hours) and occurred both during gestation and lactation suggesting no effect of accumulation of FLX/norfluoxetine levels in the blood. To confirm this, FLX and norfluoxetine levels were measured in maternal blood plasma 23 h after the first oral treatment and weekly thereafter. Subsequently, weekly FLX and norfluoxetine levels were always measured 23 h after the oral treatment, right before the new treatment, with the exception of week 3 to prevent extra stress during the week of delivery. FLX/norfluoxetine levels (FLX/NFLX in ng/ml: 23 h: 47/233; week 1: 119/729; week 2: 97/774; week 4: 89/745; week 5: 62/516; week 6: 28/442) appeared within normal range (Caccia et al., 1990; Olivier et al., 2011), indicating that there was no accumulation of FLX in blood plasma. Some dams exposed to 10 mg/kg FLX showed physical signs of toxicity, such as piloerection and lowered activity (personal observations). Clinical signs of toxicity and less increase in body weight during pregnancy have been observed before in rats as a result of a daily dose of 12 and 17 mg/kg FLX given by oral gavage during G6-G15 or G7-PND21, but dam mortality was not observed (Byrd et al., 1994; Müller et al., 2013). Perhaps, the increased toxicity in FLX treated dams may be the result of our dams being heterozygous for the SERT gene. Humans who carry the short allele of the SERT gene polymorphism, and thus have reduced SERT gene expression levels similar to SERT^+/-^ rodents, have an increased risk of developing side effects in response to SSRI treatment (Luddington et al., 2009). Whether the genotype is indeed the cause of dam mortality is something that will be further investigated in future studies incorporating wildtype, SERT^+/+^ dams.

Furthermore, FLX exposed pups had a lower birth weight, decreasing their chances of survival at birth and the first PNDs. Indeed, we saw a lower viability- and live birth index, suggesting fetal and neonatal toxicity as well. Indeed, increased mortality and lower birth weight after FLX or paroxetine exposure in pups is not uncommon (Voorhees, 1994; Fornaro et al., 2007; Cagiano et al., 2008; Van den Hove et al., 2008; Müller et al., 2013). Both human and preclinical studies show an association between SSRIs and lower birth weight (reviewed in Hutchison et al., 2018). By blocking the SERT, FLX can acutely increase serotonin plasma levels resulting in restricted umbilical artery blood flow due to serotonin’s vasoconstrictive properties (Taniguchi et al., 1994). In turn, this can lead to a reduced oxygen and nutrient supply to the fetus, thereby restricting growth. In the present study MS also reduced offspring birth weight, but did not affect pup survival. Even so, differences in birth weight were restored at adulthood, since differences were no longer present on PND70.

However, another possible cause of decreased pup survival during the first PNDs in FLX exposed litters might be due to the maternal care of the dam. Personal observations were made on maternal care on the day of birth. After delivery of pups, observed was whether pups received good maternal care by being all together and clean in the nest. When pups were found with their umbilical cord and/or placenta still attached or scattered through or outside the nest this was considered as poor maternal care. It appeared that maternal care seemed to be poor in more litters from FLX treated dams (∼80%), than in litters from VEH treated dams (∼7%). Even so, we did not score maternal care in the following PNDs and thus do not know what the quality and quantity of maternal care is for the surviving offspring. Next, the present study showed a shortening of the gestational period in dams that were stressed early in life. Similarly, maternal stress during pregnancy can lead to human pre-term delivery (Rondó et al., 2003; Lilliecreutz et al., 2016). FLX may also have influenced the maternal behavior, which in turn may have influenced the viability of the pups. In dams, serotonin metabolism seems to be altered in brain regions important in maternal behavior (reviewed in Gemmel et al., 2018). Exposing dams to FLX might have changed maternal behavior contributing to reduced viability of the pups. Indeed, maternal SSRI treatment decreases nest building in mice especially on the first PND (Svirsky et al., 2016). Unfortunately maternal behavior was not observed in the present study. Whether maternal behavior influenced the viability of the pups needs further investigation.

Overall, FLX treatment of the dams resulted in adverse pregnancy outcomes, including dam and pup toxicity, lower pup survival, reduced pup birth weight and a shorter gestational length. MS in dams had fewer adverse effects on pregnancy outcomes as it only reduced birth weight and shortened gestational length. Pups recovered from their lower birth weight as differences in body weight were no longer present at adulthood.

### Perinatal SSRI Exposure and USVs

To assess the effects of perinatal FLX exposure on total duration of USVs at PND6, pups were individually separated from the dam and littermates and USVs were recorded for 3 min. Interestingly, a profound reduction in total USV duration as a result of developmental FLX exposure in the offspring was found regardless of sex and genotype. However, this effect was only significantly present in wildtype males when looked at individual groups. These results would suggest that our FLX exposed pups are less anxious since the emission of 40-kHz USVs in response to being isolated from the nest can be seen as a direct correlate of distress and/or anxiety in the pup (reviewed in Schwarting and Wöhr, 2012).

Our results disagree with a previous study also treating rat dams with 10 mg/kg FLX that found an increase in 40-kHz USVs, and thus presumably in anxiety levels, in 10-day-old pups (Cagiano et al., 2008). However, in this study SSRI treatment was given only during the prenatal period, whereas we treated dams throughout the pre- and postnatal period. Consequently, our pups still received FLX via the breast milk of the dams on PND6, whereas the pups of Cagiano et al. (2008) were no longer exposed to FLX. Therefore, it is most likely that FLX/norfluoxetine levels in the blood plasma were present in our pups at the time of testing on PND6, explaining the decrease in USVs. Similarly, when pups are injected intraperitoneally with 0.3–30 mg/kg of the SSRI citalopram or 3 or 30 mg/kg FLX between PND8–10, the total duration of USVs was reduced compared to VEH treated pups (Hodgson et al., 2008). This is not surprising, as SSRIs exhibit anxiolytic properties in humans as well as in rodents. Altogether, our results support the hypothesis that increasing extracellular 5-HT levels results in a decrease in maternal separation-induced USVs (Wöhr and van Gaalen, 2018). Future studies should address the effects of developmental FLX exposure on USVs after the treatment period is over, and FLX/norfluoxetine is no longer present. For example, it would be interesting to combine USV recordings with juvenile social play behavior or adult social interaction to establish possible long term effects of developmental FLX exposure on social communication.

### Perinatal SSRI Exposure and Social Behavior

Exposure to SSRIs early in development can lead to short- and long-term changes in brain and behavior (Olivier et al., 2013). Although studies are conflicting, perinatal SSRI exposure in humans has been associated with increased externalizing behaviors such as aggression or defiant behavior (Oberlander et al., 2007), but also with increased internalizing behaviors such as anxiety, depression and social withdrawal (Oberlander et al., 2010; Hanley et al., 2015). More recently, multiple meta-analysis studies report an association between SSRI use during pregnancy and an increased risk for ASD in children (Kaplan et al., 2016, 2017; Kobayashi et al., 2016; Andalib et al., 2017; Brown et al., 2017), which is characterized by social interaction difficulties, communication challenges and a tendency to engage in repetitive behaviors. However, maternal illness is a confounding factor that may drive this increased risk for ASD, thus highlighting the need for animal research including both SSRI exposure and maternal stress, both separately as well as combined.

Consequently, more preclinical studies have been performed to characterize the effects of SSRIs on various forms of offspring social behavior. At the juvenile age, social play behavior in rats is essential for the development of social, cognitive, emotional and physical skills (Vanderschuren and Trezza, 2013). Typical play behavior in rats consists of rapid bouts of social interaction, which involve characteristic behaviors such as pouncing, pinning, boxing and chasing. Since the playfulness of the animal strongly depends on the social activity of its partner (Pellis and McKenna, 1992; Varlinskaya et al., 1999), both rats in a play couple were similarly treated and considered as one experimental unit. We chose this approach to emphasize possible social deficits in same treated animals, something which is done by many others, including experts in the field of social play behavior (Rodriguez-Porcel et al., 2011; Trezza et al., 2011; Khatri et al., 2014; Achterberg et al., 2015). In line with our own findings, the majority of studies find that social play behavior with an unfamiliar play partner at the juvenile age is reduced after SSRI exposure, regardless of time and length of exposure (Olivier et al., 2011; Rodriguez-Porcel et al., 2011; Simpson et al., 2011; Khatri et al., 2014). However, these SSRI-dependent alterations in play behavior can also be sex-mediated, as studies showed males were more affected (Rodriguez-Porcel et al., 2011) or no effects were found in females (Simpson et al., 2011). In the present study, we observed play behaviors to be equally reduced by FLX exposure in males and females, but the combination of MS and FLX exposure only lowered male play behavior in the offspring. Even though, not all studies are consistent in finding reduced play behavior after FLX exposure. Recently, Gemmel et al. (2017) found no effects of FLX exposure during pregnancy and lactation on behavior in offspring from non-stressed mothers. This was observed during play behavior of four familiar siblings in their home cage, and might highlight the importance of a novel play partner to observe effects of FLX. Interestingly, when Gemmel et al. (2017) looked at social play behavior with an unfamiliar play partner, they found that FLX exposure resulted in an increase in play behaviors in these animals. This specific effect on play behavior at the juvenile age in rats has not been observed by us or by others (Olivier et al., 2011; Rodriguez-Porcel et al., 2011; Simpson et al., 2011; Khatri et al., 2014).

Previous findings on more general social interaction behavior at an adult age, such as social exploration or contact behaviors, are more conflicting. In the present study, an interaction was found between FLX exposure, genotype and sex. FLX exposure during the entire gestational period and lactation reduced the total time spent in social interaction in adult male, but not female offspring. In fact, female adult social interaction was not affected at all by FLX exposure, suggesting increased vulnerability to developmental FLX exposure in males. Olivier et al. (2011) similarly found that FLX treatment of the dams during pregnancy tends to reduce social exploration in adult male offspring. In contrast, male rat offspring directly injected with FLX from PND0–4 increased their sniffing, contact and total interaction behavior with a conspecific when adult (Ko et al., 2014). This could be due to compensation for impaired tactile function, as seen by structural alterations in the barrel cortex after SSRI exposure (Lee, 2009). Other studies investigating FLX exposure during the entire pregnancy, or part of the gestational and lactation period, found no effects on adult male and female social exploration in mice, but did find an increase in male aggressive behavior (Kiryanova and Dyck, 2014; Svirsky et al., 2016). Overall, studies exploring the effects of SSRIs on adult social interaction in rodents are inconclusive, and effects on females are underexposed.

Next to looking at social exploration in rodents, another way to assess adult social behavior is to study the motivation for interaction with a conspecific over interaction with an object. The majority of such studies find decreased motivation for social interaction with a conspecific after developmental SSRI exposure in both sexes (Rodriguez-Porcel et al., 2011; Simpson et al., 2011; Khatri et al., 2014; Zimmerberg and Germeyan, 2015). Even so, a study in mice found an increase in motivation for conspecific interaction, but in females only (Svirsky et al., 2016).

Because we did not investigate the brains of the offspring we can only speculate on the effects developmental FLX exposure had in brain regions important for social behavior. It was previously found that the size of the sexually dimorphic nucleus of the medial preoptic area was decreased in male offspring exposed to postnatal FLX (Rayen et al., 2013). Although this area is linked to sexual behavior and not *per se* to social play behavior or social interaction, it was an effect prominently found in males, indicating that alterations in brain regions after developmental SSRI exposure can be sex-dependent. Furthermore, perinatal SSRI treatment is able to reverse hippocampal CA3 spine reduction and synapse density in juvenile and adolescent males that were prenatally stressed (Ishiwata et al., 2005). The CA3 region is linked to the CA2 region (Sekino et al., 1997), an important area for regulating social behavior (reviewed in Gemmel et al., 2018). As such, these alterations may reflect changes in social behavior outcomes when rats are exposed to FLX and/or stress. Alterations were also found in the dendritic spine architecture of the basolateral amygdala when exposed to postnatal FLX treatment. Interestingly this was linked to increased social behavior in male offspring. As mentioned before studies in females are limited, especially when combined with maternal stress. Both stress and SSRI treatment may have independent, but also accumulating effects, on brain development. Further investigation is warranted to unravel these alterations and to investigate their relevance in social functioning later in life in both males and females.

Altogether, the present and previous studies show that FLX exposure during early development interferes with juvenile social play behavior and social interaction during adulthood with males appearing to be more vulnerable to these effects than females.

### Maternal Stress, Perinatal SSRI Exposure, and Offspring Social Behavior

Since SSRI treatment occurs in patients with a major depression and not in healthy people, using an animal model for maternal depression in the form of early life stress in SERT^+/-^ dams might be of high translational value. Preclinical studies investigating SSRI exposure in an animal model of maternal stress are limited in number, especially when looking specifically into the effects of such developmental exposure on social behavior on the short and long-term. In the present study, few effects of MS were found on offspring social behavior. MS only tended to reduce total play behavior in female offspring regardless of FLX treatment and had no effects on male play behavior. Previously, Gemmel et al. (2017) used an animal model of pre-gestational stress and found decreased juvenile sibling play behavior regardless of sex, but not when playing with a novel play partner. However, in the same study an increase in time spent grooming a novel conspecific was found. Interestingly, we found a similar increase in adult social exploration in the offspring due to MS, but only in female wildtype offspring.

There are only few studies that preceded us in using an animal model of maternal stress to investigate effects of SSRI exposure during development. In the study of Gemmel et al. (2017) FLX exposure rescued the observed reduction in sibling play behavior as a result of pre-gestational stress exposure alone, regardless of sex. Interestingly, in the present study, the combination of MS and FLX exposure showed no such effects in the offspring, since there was no lowered social play due to MS that could be rescued. Instead, the combination of MS and FLX exposure resulted in the same play reducing effects as when exposed to FLX alone in male offspring. In juvenile female offspring, MS together with FLX exposure no longer resulted in the reduced social play behavior as seen when exposed to FLX alone. However, the combination of MS and FLX exposure seemed to increase adult play behavior in the offspring compared to offspring from early life stressed dams treated with VEH, while total social behavior in males similarly increased compared to offspring from CTR dams treated with FLX. Even so, this effect was only found in SERT^+/-^ male offspring.

The limited effects of MS on offspring social behavior found in this study might be surprising, since maternal stress can result in long-term changes in affective behavior in the next generation (Franklin et al., 2010). One explanation could be that the maternal stress applied in the present study only leads to subtle changes in social behavior and for that reason these changes could not be detected in the current social tests. Although anhedonia was present in the dams, other affective behaviors were not changed relative to CTR dams (Houwing et al., 2019). Even though we applied a more severe stressor to the mothers compared to other maternal separation studies with SERT^+/-^ rats (Houwing et al., 2017), this still might be too mild to find robust effects on social behavior in the offspring. Future studies are needed to explore whether even more severe stressors, e.g., like the unpredictable maternal separation used by Franklin et al. (2010), will result into changes in social behaviors in the offspring.

A limitation of the current study is that effects found on social behavior due to FLX exposure during development could be mediated by the effects of maternal behavior. Since we only made personal observations on maternal care on the day at birth and not afterward, we cannot rule out whether maternal behavior was affected, and thereby influencing social behavior of the offspring. FLX treatment of the dams can increase maternal care of the pups (Johns et al., 2005; Pawluski et al., 2012), but other studies find no effect on maternal behavior (Rayen et al., 2011). Maternal licking, a component of maternal behavior, is negatively correlated with social play behavior with an unfamiliar play partner (Gemmel et al., 2017). To know whether this was the case in the present study, maternal care with and without FLX treatment should be subjectively observed for a longer period in our early life stressed dams in the future, especially since maternal toxicity was observed.

### Offspring SERT Genotype and Social Behavior

To our knowledge, this is the first study that explored perinatal SSRI effects on social behavior in rat offspring including SERT genotype of the offspring as well. Although no differences in social play were present at the juvenile age, differences were found in adult social interaction between SERT^+/-^ and SERT^+/+^ animals. Overall, SERT^+/-^ males and females show reduced social exploration relative to wildtypes, regardless of MS and FLX exposure. Again, effects were more pronounced in male offspring than in female offspring, reducing social exploration in all offspring groups, whereas this was only found in female offspring from early life stressed dams that were VEH treated. Similarly, total social behavior in SERT^+/-^ males was reduced in all offspring groups compared to SERT^+/+^ animals, except for SERT^+/-^ offspring from early life stressed dams treated with FLX. No genotype effects were observed on total social behavior in females. Previously, studies have shown that SERT^-/-^ rodents are less interested in socially exploring or playing with a conspecific (Homberg et al., 2007b; Kalueff et al., 2007; Schipper et al., 2011a,b), but social behavior studies including SERT^+/-^ rodents are limited. When they do include SERT genotype, social behaviors such as sexual behavior and aggression are not affected in SERT^+/-^ rodents compared to SERT^+/+^ animals (Homberg et al., 2007a; Chan et al., 2011). When it comes to investigating differences in SERT genotype in response to FLX treatment, studies are limited in number. Since little neurochemical and behavioral changes have been observed in SERT^+/-^ rodents when compared to SERT^+/+^ animals, we similarly did not expect to see a difference in response to FLX exposure during development between genotypes. The few studies that took SERT genotype into account when looking at perinatal FLX exposure found no differences between SERT^+/+^ and SERT^+/-^ mice in response to FLX exposure in anxiety-related tests (Ansorge et al., 2004).

However, we found that adult SERT^+/+^ offspring from dams treated with FLX reduced social behavior compared to offspring from VEH treated dams, while SERT^+/-^ offspring did not. Noted should be that it also looked like SERT^+/-^ offspring reduced social behavior after FLX exposure, but this did not reach statistical significance. Overall, the present study is the first to show differences in specific adult social behaviors between SERT^+/+^ and SERT^+/-^ animals, indicating that reduced SERT expression can interfere with social behavior during adulthood.

In light of recent findings associating perinatal SSRI treatment with impaired social behavior in children, there is need for preclinical research exploring the effects of SSRIs and maternal stress both independently as well as combined. In the present study, MS and FLX exposure interacted to affect aspects of offspring adult social behavior in males, thus highlighting the importance of implementing an animal model of maternal stress in SSRI exposure research. In addition, our findings suggest that males may be more vulnerable to perinatal FLX exposure, since FLX induced effects on female adult social behavior were absent.

However, since studies vary in their protocols on many levels (differences in species, test age, drug dose, drug administration route, duration of drug exposure, exposure during the pre- or postnatal period, and differences in social behavior paradigms), replication of findings is of great importance. Replicating findings despite differences in protocols will further support observed outcomes. Here we showed in two different social behavior paradigms, one at a young age and the other in adulthood, that social behavior was affected by perinatal FLX exposure. Since social experience can influence the behavioral outcome, we did not repeat the exact same social behavioral tests at young age and adulthood. Currently, our group is also addressing other social behaviors including aggression and sexual behavior to provide a better insight into the effects perinatal FLX exposure and/or early life maternal stress can have on the social outcome of the offspring. Furthermore, when it concerns social behavior it becomes especially important to include both male and female offspring, as boys are more often diagnosed with social disorders. Findings in males may therefore not be representative for females. Overall, investigating the association between perinatal SSRI exposure and offspring social behavior can contribute to extending our knowledge about the risks and/or benefits of perinatal SSRI treatment for the mother and the developing child.

## Author Contributions

JO and DH designed the study. DH, LS, JS, and AR performed the experiments. DH performed the statistical analyses and drafted the manuscript. JO, AR, MW, and SdB critically revised the manuscript. All authors have finalized and approved the content of the manuscript.

## Conflict of Interest Statement

The authors declare that the research was conducted in the absence of any commercial or financial relationships that could be construed as a potential conflict of interest.

## Abbreviations

ANOVA: analysis of variance
CTR: control
FLX: fluoxetine
MS: maternal early life stress
PND: postnatal day
SERT: serotonin transporter
SSRI: selective serotonin reuptake inhibitor
USV: ultrasonic vocalization
VEH: vehicle
