# Supplementary material for: Subjecting Dams to Early Life Stress and Perinatal Fluoxetine Treatment Differentially Alters Social Behavior in Young and Adult Rat Offspring
Source: Front Neurosci. 2019 Mar 12;13:229. doi: 10.3389/fnins.2019.00229 (PMC6423179; doi:10.3389/fnins.2019.00229)
Supplement: Supplementary file 1 [file Table_1.DOCX]

Supplementary table 1. 4-Way (Maternal Early Life Stress x Fluoxetine x Genotype x Sex) ANOVAs

|  | **Behavior** | **Main Effect of Maternal Early Life Stress (MS)** | **Main Effect of Fluoxetine exposure (FLX)** | **Main Effect of  Genotype** | **Main Effect of Sex** | **Interaction Effect** |
| --- | --- | --- | --- | --- | --- | --- |
| **Social Play** | | | | | | |
|  | Social exploration (sec) | n.s. | F_(1,167)_=9.526, p=.002 | n.s. | n.s | MS x FLX x Genotype, F_(1,167)_=8.879, p=.003 |
|  | Pouncing (freq) | n.s. | F_(1,167)_=18.681, p<.001 | n.s. | n.s. | n.s. |
|  | Pinning (freq) | n.s. | n.s. | F_(1,167)_=3.947, p=.049 | n.s. | MS x FLX x Genotype, F_(1,167)_=5.561, p=.020 |
|  | Chasing (sec) | n.s. | F_(1,167)_=15.638, p<.001 | n.s. | n.s. | MS x Sex, F_(1,167)_=11.066, p=.001 |
|  | Total play (sec) | n.s. | F_(1,167)_=10.705, p=.001 | n.s. | n.s. | n.s. |
| **Social Interaction** | | | | | | |
|  | Social exploration (sec) | n.s. | n.s. | F_(1,160)_=32.616, p<.001 | F_(1,160)_=40.764, p<.001 | n.s. |
|  | Follow/approach (sec) | n.s. | n.s. | n.s. | n.s. | n.s. |
|  | Play Behavior (sec) | n.s. | n.s. | n.s. | F_(1,160)_=45.186, p<.001 | MS x FLX, F_(1,160)_=7.664, p=.006 FLX x Genotype x Sex, F_(1,160)_=5.757, p=.018 |
|  | Total social behavior (sec) | F_(1,160)_=3.975, p=.048 | n.s. | F_(1,160)_=20.261, p<.001 | F_(1,160)_=110.660, p<.001 | FLX x Sex, F_(1,160)_=6.310, p=.006 FLX x Genotype x Sex, F_(1,160)_=51.283, p<.001 |
